# Structural Sensitivity of N 1s Excitations in *N*-Methylacetamide Solutions

**DOI:** 10.1021/acs.jpclett.4c03487

**Published:** 2025-02-06

**Authors:** E. A. Eronen, A. Vladyka, Ch. J. Sahle, J. Niskanen

**Affiliations:** † University of Turku, Department of Physics and Astronomy, FI-20014 Turun yliopisto, Finland; ‡ ESRF, The European Synchrotron, 71 Avenue des Martyrs, CS40220, 38043 Grenoble Cedex 9, France

## Abstract

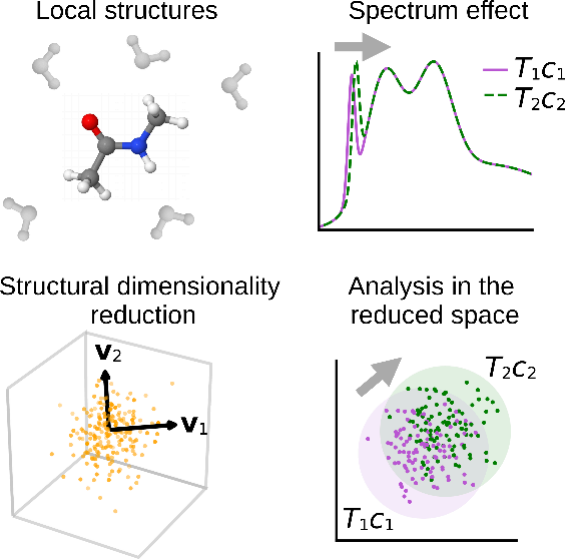

Interpreting the X-ray spectra of liquids is complicated by their selective structural sensitivity and ensemble averaging. We report nitrogen K-edge spectra of liquid *N*-methylacetamide and its water solutions at temperatures of 305 and 350 K. The pre-peak in the spectrum shows a shift with an increase in the temperature or *N*-methylacetamide concentration. The effect is reproduced by our classical molecular dynamics simulations and subsequent spectrum calculations using density functional theory. We apply a data-driven method, emulator-based component analysis, to the computational data to identify the decisive structural degrees of freedom behind spectral variation. This representation in reduced dimensions accounts for the involved loss of structural information and reveals that the effect is indicative of weakening of the hydrogen bonds.

Interactions between molecules cause a liquid system to have a complex potential energy surface characterized by numerous local energy minima. Consequently, the system is not well represented by a single configuration, but explaining an experiment requires sampling for the ensemble average. Interestingly for the context, X-ray spectra of different local structures **R** in the accessible phase space have been found to vary drastically,^1−4^ with differences potentially larger than those observed in ensemble averages of, e.g., different phases.^3^ This raises the question of what structural conclusions are implied by changes in such spectra, especially if they are sensitive to only some of the numerous structural characteristics. Because of advances in computational resources and simulation methods, machine learning (ML) can now provide an answer. In the stated problem, it is useful to consider the entire structure–spectrum relation and separate the decisive structural features from the rest. Such a division can be achieved by, e.g., a recently introduced emulator-based component analysis (ECA),^5,6^ a decomposition method based on ML and guided by the spectral response.^4,5,7,8^ As a result of this projection pursuit algorithm, a spectrally relevant structural subspace is obtained. Successful ML and ECA decomposition requires a descriptor **D**(**R**) of a structure instead of the raw atomic coordinates **R**. When combined with a human-interpretable descriptor, like a local version of the many-body tensor representation^9^ (LMBTR), the results of ECA become human-interpretable.

In this work, we report and analyze a temperature- and solvent-dependent effect at the pre-peak of nitrogen K-edge spectra of liquid *N*-methylacetamide [NMA (Figure 1)] and its water solutions. Although our work is motivated by the structural information content of ensemble-averaged spectra, this particular molecule is interesting also because it contains a peptide bond-like arrangement and is a potential solvent for amino acid chains.^10^ Density functional theory (DFT) simulations on structures sampled from classical molecular dynamics (MD) reproduce the experimental effect, which we analyze further using ECA. For this, we train a neural network (NN) to predict intensities in spectral regions from the corresponding structures using a data set sampled to uniformly cover the entire available structural space. The analysis procedure reveals that the spectral effect is mainly induced by weakening of hydrogen bonds with an increase in temperature or NMA concentration.

**Figure 1:**
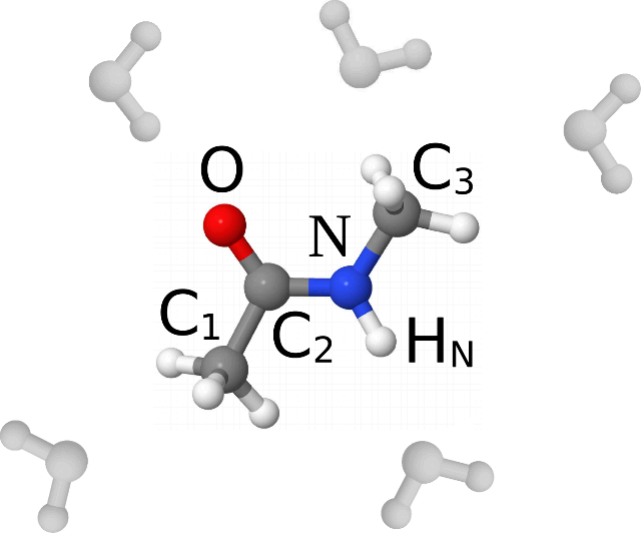
Schematic of the NMA molecule with labeling of the atoms of interest. The water solvent is colored gray. The plot was prepared with Jmol.^11^

Figure 2 shows the nitrogen K-edge hard X-ray Raman scattering (XRS) spectra for the measured concentrations *c* at temperatures *T* = 305 and 350 K. We study pure liquid NMA and its water solutions at NMA/H_2_O molecular number ratios (MNRs) of 0.33, 0.25, and 0.20. The spectra consist of three main features, starting with a pre-peak at ∼402 eV, which is rather similar to that observed in the gas phase for the N 1s  excitation at the “peptide” bond.^12,13^ The other features are a main edge at ∼407 eV and another peak near the computed N 1s binding energy at ∼413 eV. Our previous experiment with the nitrogen K edge of aqueous triglycine^4^ also produced a similar pre-peak as shown here, but there was no second peak after the main edge.

**Figure 2:**
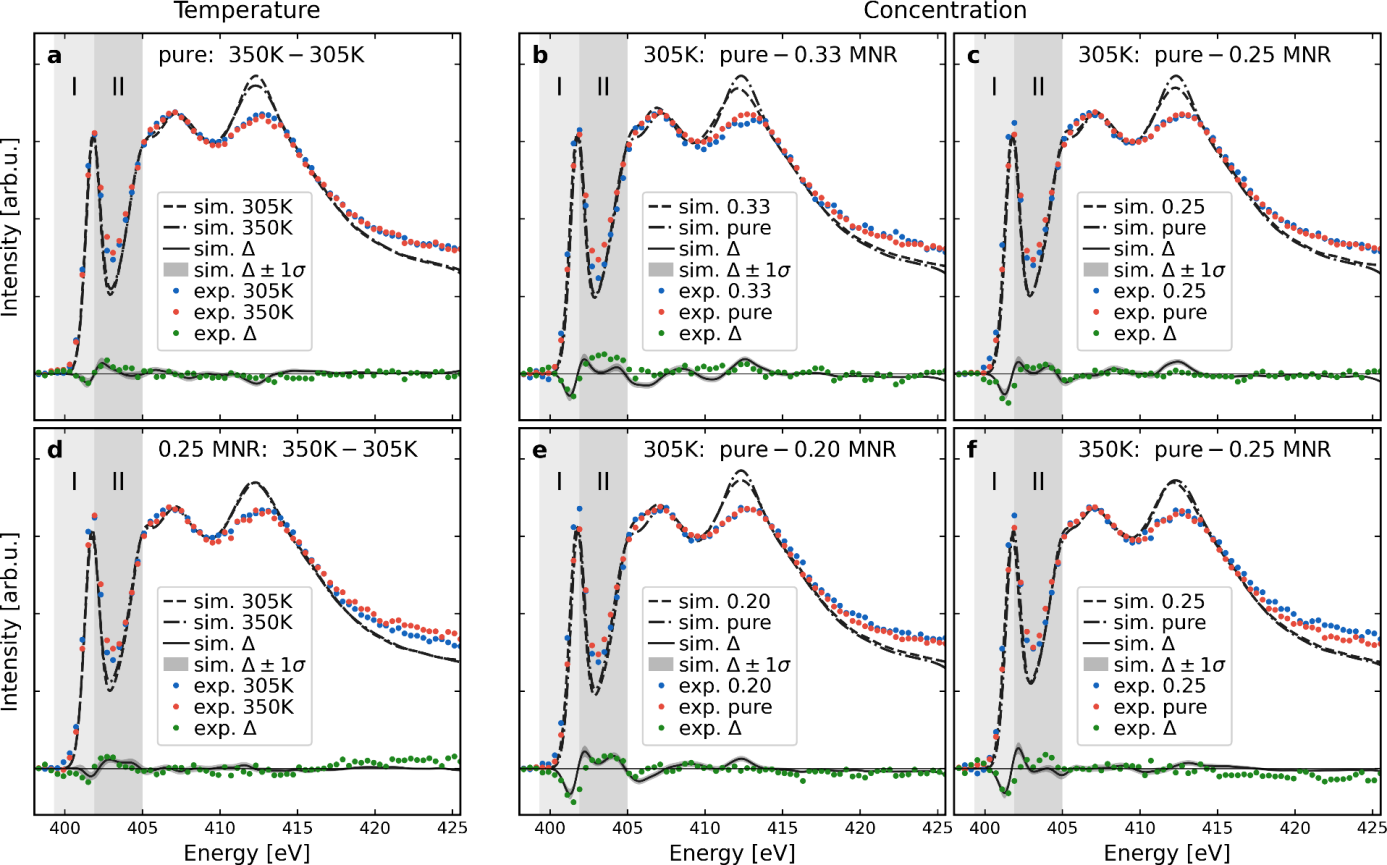
Experimental X-ray Raman scattering spectra and computational ensemble-averaged X-ray absorption spectra for liquid *N*-methylacetamide and some of its water solutions. (a and d) Temperature dependency via experimental and computational difference profiles Δ between 350 and 305 K systems of pure NMA and 0.25 MNR mixture. (b, c, e, and f) Comparison between spectra of pure NMA and an aqueous solution. Statistical uncertainty of the difference profiles is calculated with a 100 000-fold bootstrap and denoted with Δ ± 1σ. We define two regions of interest (ROIs), I and II, from the *T*- and *c*-dependent effect to be used in further analysis.

The experiment shows a *Tc*-dependent effect at the pre-peak, visualized by difference profiles in Figure 2. The intensity shifts to higher energies both with an increase in temperature *T* and upon removal of the water solvent. The figure also shows that this phenomenon is reproduced by our simulated ensemble average X-ray absorption spectra, each of which was computed from 400 individual local structures randomly sampled from MD trajectories. For detailed analysis of the indicated structural changes behind the spectral effect, we define two regions of interest (ROIs, I and II) based on the difference profile at the pre-peak region. To give both equal importance in the analysis, we study the total areas within the computational ROIs after applying *z*-score standardization. This transformation ensures that the distribution of data points for each feature across the entire data set has a mean of zero and a standard deviation of one, bringing the two different feature data sets onto comparable scales.

Our analysis requires an ML emulator capable of predicting the two spectral ROIs for a local structure. Therefore, we needed a training data set, the size of which was limited by heavy quantum-mechanical spectrum simulations. As the computationally light classical MD simulations produced plenty of data, we first carried out disperse sampling of MD structures, for which we then evaluated the spectra. This method for a wide and uniform structural spread is analogous to farthest point sampling^14^ and aims for the efficient allocation of resources. We encoded structural data **R** with an LMBTR descriptor **D**(**R**) using an in-house implementation inspired by the DScribe package.^15,16^ The descriptor consisted of element-wise Gaussian-smeared distance distributions for five center atoms of the absorbing NMA molecule: N, C_2_, C_3_, H_N_, and O as depicted in Figure 1. For the emulator, we used a fully connected feed-forward NN implemented in PyTorch version 2.2.1^17^ on *z*-score-standardized **D**(**R**) → **D̃**(**R**) structural data. The NN and LMBTR contain numerous hyperparameters to tune. These include, e.g., the width, the depth, and the weight decay parameter of the NN, and the spatial grid of the descriptor. As each of the hyperparameters has a wide range of possible values, we ran a grid search for them as described in ref (8) and chose the best-performing NN–LMBTR combination. The final model yielded an *R*^2^ score of 0.808 on a test set consisting of structure–ROI pairs representing the statistical ensembles for each *Tc* point. We use the *R*^2^ score for performance metrics throughout this work owing to its analogy to principal component analysis but note that any other reasonable score could be applied. For more details about the ML, see the Supporting Information.

We fitted two ECA vectors on the training set, maintaining a majority of the spectral variance with an *R*^2^ of 0.752 on the test set. Thus, the structural dependence of ROIs I and II can be almost completely encoded into latent variables *t*_1_ and *t*_2_ by projection on the corresponding structural basis vectors **v**_1_ and **v**_2_. This procedure serves as dimensionality reduction of **D**(**R**) with the maximal explained ROI intensity variation. Gaussian-smoothed (full width at half-maximum of 5) two-dimensional histograms of the latent variables, representing the statistical ensemble, are shown in panels a and e of Figure 3 for pure NMA. In terms of the observed spectral effect, the difference between the histograms provides an informative representation of the changes in the spectrally relevant subspace. These results are shown in panels b–d and f–h of Figure 3 and indicate notable shifts in the distribution between two *Tc* points. The *t*_1_ value always tends to decrease with an increase in temperature *T* and with removal of water, following the spectral effect. Latent variable *t*_2_, less spectrally significant, does not show behavior that is as consistent.

**Figure 3:**
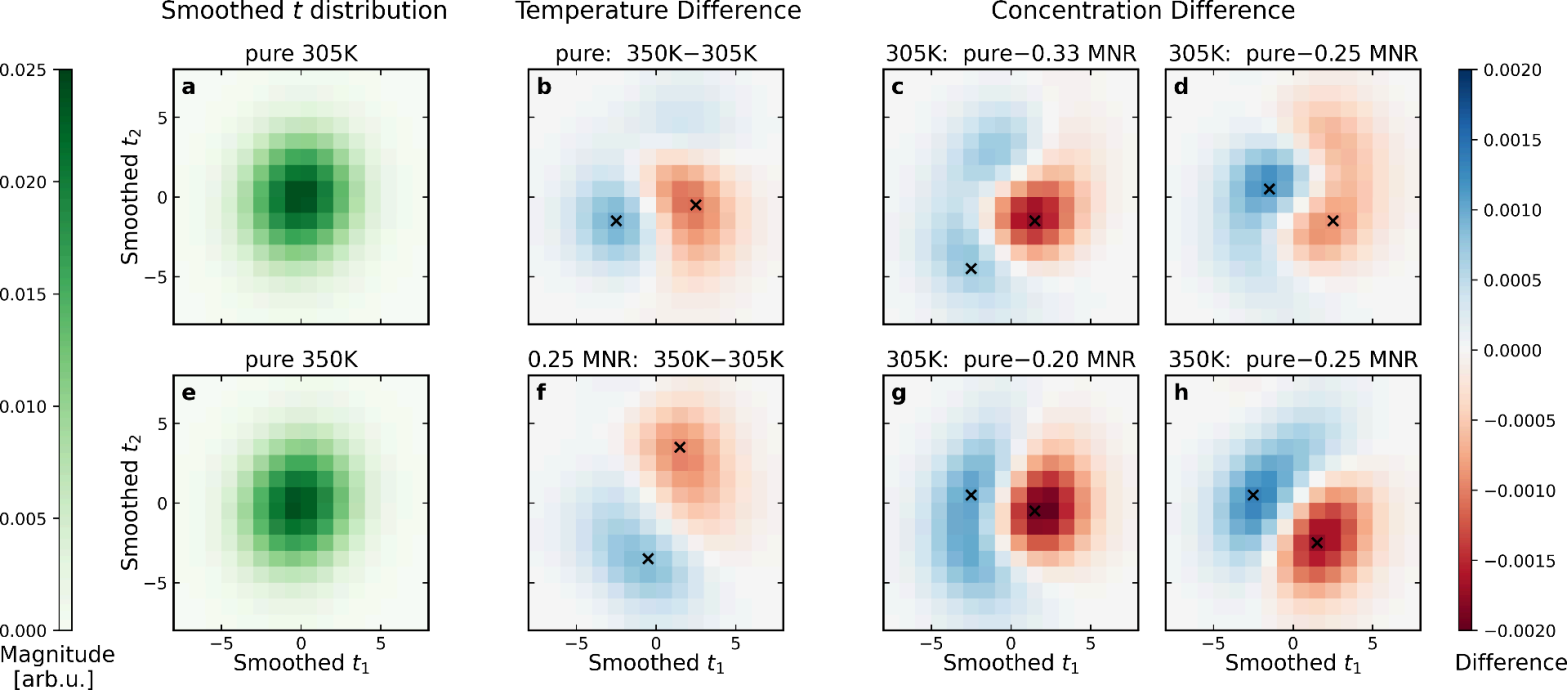
(a and e) Smoothed structural feature distributions in reduced dimensions of eq 1 for pure NMA. Corresponding differences between the distributions for (b and f) temperature *T* and (c, d, g, and h) *c*. All of the difference histograms show a shift in the spectrally decisive structural distributions. Notably, *t*_1_ always tends to decrease when temperature *T* or *c* is increased. The extrema of the difference histograms, used in further analysis, are denoted with crosses.

For a concrete structural interpretation of the effect in the pre-peak, we select the extrema of the aforementioned difference distributions, marked with crosses in Figure 3, and perform a two-dimensional ECA expansion for them. This gives rise to approximate structures
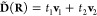
1the differences of which are shown in Figure 4 after inverse transformation **D̃**(**R**) → **D**(**R**) to the absolute scale. In addition, the curves have been divided by the square of the according distance, resulting in curves for the radial number density with radial behavior proportional to that of the respective radial distribution function. The non-zero values of this plot indicate structural changes between the two *Tc* points that are implied by the spectra.

**Figure 4:**
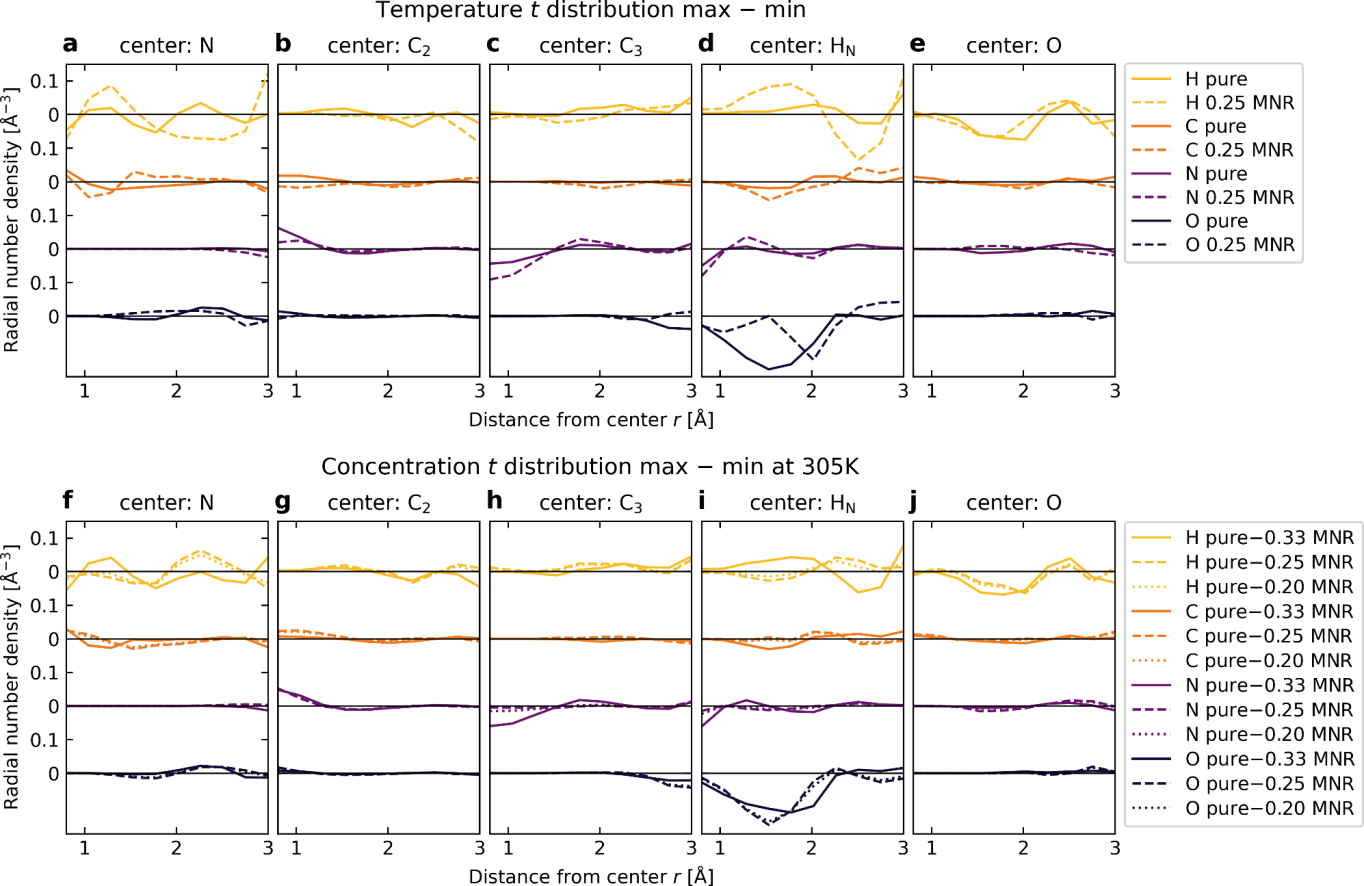
Analysis of the decisive structural shifts behind the spectral effect. Differences between two-component ECA expansions **D**(**R**) of the maximum and minimum points in the difference histograms of Figure 3. (a–e) Changes in structure upon heating from 305 to 350 K. (f–j) Change in structure at 305 K with an increase in NMA concentration. Panels (d and i) indicate a change to fewer oxygen atoms at ∼1.5 Å. Values near zero indicate structural features with little to no effect on spectral ROI intensities. The expansion has been divided by the squared distance to the corresponding center to allow comparison with the radial distribution function. See the text for details.

From this analysis, the *T*- and *c*-dependent effects seem to be qualitatively similar. Generally, the H_N_–O curves of panels d and i of Figure 4 show the largest of the effects at ∼1.5 Å. This feature is characteristic of the nearest O neighbor (LMBTR broadening σ = 0.4 Å) and can therefore be attributed to hydrogen bonds becoming weaker or breaking as the temperature or NMA concentration is increased. Changes in hydrogen bonding may also be reflected by the H_N_–H and O–H curves, but detailed analysis is more cumbersome due to both the absorbing NMA molecule and the solvent containing numerous hydrogen atoms. Some minor contributions can also be traced to changes in the distances between neighboring atoms within the NMA molecule. Next, we turn to a discussion of these results.

Instead of using raw atomic coordinates **R**, descriptors **D**(**R**) are commonly employed to represent structural information in a form that is easily digestible for an ML model. Numerous encoding methods can provide an accurate emulation for X-ray spectra,^8^ but their typical problem is poor interpretability by a human. Our previous works with similar systems^4,8^ have found LMBTR to be a capable descriptor with human-interpretable properties, namely the two-atom element-wise broadened distance distributions. In this work, we found that a descriptor that performs well can be built by calculating these distributions for multiple center atoms. Moreover, this fully interpretable descriptor appears to excel in ECA decomposition, where the goal is to maximize covered spectral variance with minimal degrees of freedom. We discovered that all of the necessary structure–ROI intensity information lies within a 3 Å radius of the center atoms, as no performance gains were observed by extending this limit in the model selection. Although the descriptor is highly interpretable, we note that the Gaussian broadening may cause the features to deviate from the true displacement, a fact that has to be considered when analyzing the ECA component vectors and ECA expansion.

Random sampling of MD structures inevitably favors regions of phase space associated with a low free energy. While this sampling method is well suited for estimation of the ensemble average, it is likely a sub-optimal choice for creating a training data set for an ML model. In fact, a model trained on randomly sampled data has shown a declining spectrum prediction performance as data points deviate farther from the minimum energy structure.^18^ Because each of the data points weighs equally in the cost function during the training phase of ML, it is reasonable to expect that a uniform distribution of data would avoid selection bias toward specific regions and lead to an improved overall generalizability of the model. Additionally, the data set should cover the structural space as broadly as possible to avoid extrapolation, which, by analogy to curve fitting, is known to be prone to large errors. Disperse sampling of structures prior to spectrum evaluation provides a more advantageous training set on a large pool of MD structures (see the Supporting Information). This procedure generally outperforms random sampling for the spectrum prediction of the H_2_O molecule, especially when the data set is small. Disperse sampling was also less sensitive to changes in the selected training data set and generalized better in the sparse regions of the data cloud. Furthermore, finding an optimal structural sampling before running the computationally heavy spectrum calculations can be extremely beneficial by enabling efficient allocation of the available computational resources.

Studying the structural causes behind the spectral effect in liquids is complicated due to inherent structural variation. We found this variation between local structures of an NMA system to be significantly broader than the differences between the respective ensemble averages. However, only some structural changes affect the spectrum as quantified by our previous works on liquid systems.^4,8^ A method, such as ECA, is required to filter out the structural properties that are irrelevant due to the involved loss of information. As an example of this information bottleneck, the Supporting Information shows two significantly different structures with nearly equal spectrally relevant components. In accordance, the ROI intensities for these two structures are similar.

On the contrary, the revealed decisive structural characteristics yield a formalism for structural interpretation of the spectra. In ECA, the structural data points are projected onto orthogonal vectors, which are iteratively optimized one by one to maximize the emulator performance on the projected points. A fast numerical emulator, such as an NN, is a prerequisite due to the numerous approximate spectrum evaluations required. In this sense, ECA found causal connection between the observed spectral effect and changes in hydrogen bonding. This conclusion is reasonable in the sense that the similar pre-peak feature of water has also been widely considered to be sensitive to hydrogen bonding.^19^

The validity and accuracy of the ECA method are tied to several factors. First, the accuracy of the simulations is limited by the necessary approximations. We mitigated the associated risk by focusing on ROIs, the intensities of which reproduce an experimental effect. Second, the amount of available data and its spread within the structural space are restricted, which together with limited model selection cause imperfect emulator performance. Third, the structural descriptor faces several requirements. It must provide (i) accurate ML emulation and (ii) efficient ECA decomposition and (iii) be interpretable by a human.^8^ Fourth, a higher-rank decomposition would increase the covered spectral variance. However, this would happen with diminishing gains and would also add complexity to the analysis. Despite these factors, the applied method provides a mathematical framework for the analysis of the complex spectrum – structure inverse problem via a significant reduction in dimensionality.

To summarize, the observed spectral effect is mostly indicative of the weakening of hydrogen bonds as the temperature or NMA concentration increased. This conclusion is based on computations and dimensionality reduction into the spectrally decisive subspace of two structural degrees of freedom, which explain the majority of the spectral variance. In this representation, the spectral effect implies a shift in the statistical distribution of the local atomistic structures.

## Methods

The NMA sample was sourced from Sigma-Aldrich (purity of ≥99.0%), and the solutions were prepared by mixing NMA with deionized water (milli-Q, resistivity of ≈18 MΩ cm). The studied NMA/H_2_O molecular number ratios of 0.33, 0.25, and 0.20 correspond to 57.5%, 50.3%, and 44.7% of NMA (w/w), respectively, and the lower temperature of 305 K is slightly above the melting point of NMA at ambient pressure. The experiment was carried out using the multielement XRS endstation^20^ at beamline ID20 of the European Synchrotron Radiation Facility (ESRF). We used small scattering angles in the forward direction where the XRS signal is equivalent to the X-ray absorption spectrum (XAS).^21^ To minimize the radiation damage and to control the temperature of the sample, we circulated the sample in an improved version of a liquid flow cell introduced in ref (22). Additionally, we also measured the oxygen K edge of pure NMA at 305 and 350 K, for which we refer the reader to the Supporting Information.

For background removal, we used a computational background from a modified version of eq 6 from ref (23) (shifted by −10 eV) with an additional linear component. The resulting background function was fitted to the experimental spectra by minimizing the squared error (observed − fit)^2^ for energy losses in the spectral regions of 396.5–399.5 and 488–525 eV simultaneously. The fitting background consisted of contributions from hydrogen 1s, carbon 1s, 2s, and 2p, nitrogen 1s, 2s, and 2p, and oxygen 2s and 2p weighted by the number of each atom in each mixture. Next, the fitted background, without contributions from nitrogen 1s, was subtracted from the experiment. Finally, the spectra were normalized using the mean of the five points highest in intensity within the range of 405–410 eV at the main edge.

We ran MD simulations and evaluated spectra on sampled local structures. We simulated a total of four concentrations *c*: pure NMA and aqueous NMA solutions at MNRs of 0.33, 0.25, and 0.20, each at two temperatures (305 and 350 K). We utilized the OPLS/AA force field^24^ without constraints for *trans*-NMA, using parametrization obtained from Caleman et al.^25,26^ For water molecules, we used the rigid TIP4P^27^ model. For each *c*, the initial configuration was prepared in a cubic box with a volume of ≈50^3^ Å^3^. We used periodic boundary conditions, a van der Waals cutoff of 1.1 nm with dispersion corrections (to pressure and potential energy) for the constant-*NPT* simulations, and particle-mesh Ewald (PME) summation for the Coulomb interaction with a switching distance of 1.1 nm. After the initial thermalization using v-rescaling thermostating^28^ and Berendsen barostating,^29^ we ran simulations using Nosé–Hoover thermostating^30,31^ (τ = 1.0 ps) and Parrinello–Rahman barostating^32,33^ (τ = 5.0 ps, compressibility of 5 × 10^–5^ bar^–1^) for 11 ns with the time step of 0.5 fs. Thermalization in terms of temperature and density was observed well before 1 ns in these runs. These MD simulations were performed on a cluster computer using GROMACS (version 2021.6).^34^

We used the last 10 ns of each MD run for sampling of local structures **R**, which consisted of the absorbing molecule and (i) water molecules fully within the radius of 6.5 Å of any atom of the absorbing molecule and (ii) NMA molecules fully within the radius of 8.5 Å of any atom of the absorbing molecule (for details, see the Supporting Information). For training the ML model and fitting the ECA component vectors, we applied disperse sampling to pick 10 000 structures from a pool of 1 280 000 local neighborhoods with an equal number of data points from each MD run using the L_2_ norm as a distance metric for LMBTR-encoded structures. Similarly, we created a separate dispersely sampled set of 1000 points from a separate pool of 102 400 local structures for determining the early stopping condition of ML training. For testing the ML model, plotting the ensemble averages, and studying the structural shifts behind the spectral effect, we randomly sampled 400 local neighborhoods for each *Tc* point excluding data points of the previous sets. Details and justification of the procedure are presented in the Supporting Information.

We calculated the nitrogen K-edge XAS for all of the sampled structures using GPAW version 22.1.0^35−37^ and the projector-augmented wave method (PAW)^36^ for DFT. The calculations utilized the Perdew–Burke–Ernzerhof (PBE) exchange-correlation potential^38^ together with the plane wave basis with an energy cutoff value of 400 eV (for validation, see the Supporting Information). We applied the transition potential half-hole (TP-HH) approximation^39^ and evaluated excitations from the absorption site to the lowest 3500 valence single-electron states. The energy scale was corrected by using the Δ-DFT method for the lowest excitation. Using knowledge from our previous work,^4^ we added an occupation smearing with the Fermi–Dirac distribution with a width of 0.25 eV to aid convergence. In addition, a 3.0 Å vacuum was added around each snapshot of a local structure. Using 20 central processing units (CPUs) and approximately 100 gigabytes of memory for each calculation, the spectrum simulations consumed >1 million CPU hours on Intel Xeon Gold 6148 processors at a computing cluster.

The spectrum simulations produced 3500 energy–intensity pairs for each structure, which we convolved using Gaussian functions. The full width at half-maximum of these functions equals 0.92154 + max[0, 6.67 × 10^–3^(*E* – *E*_B_)] in electronvolts, with N 1s electron binding energy *E*_B_ evaluated separately for each structure. Thus, below *E*_B_ the width is constant, whereas above *E*_B_ the width increases linearly. These general values are based on fitting the sum of the ensemble mean spectra to the sum of their experimental counterparts for the best overall match for all of the data. Additionally, the energy scale of the computational results was shifted −1.15 eV. In this scale, the mean of *E*_B_ was 413.9 eV for the whole set of ensemble-averaged data, and the standard deviation of its distribution was 0.4 eV.

## Data Availability

The data and relevant scripts of this work are available at https://doi.org/10.5281/zenodo.14216288.

## Supporting Information Available

Experimental oxygen K-edge X-ray Raman scattering spectra, validation of the structural and spectral simulations, details on the disperse sampling, including an evaluation of a separate data set, details on the machine learning hyperparameters and the LMBTR descriptor, and additional results obtained with the ECA method (PDF)
Transparent Peer Review report available (PDF)
