# Supplementary material for: Structural Sensitivity of N 1s Excitations in N-Methylacetamide Solutions
Source: J Phys Chem Lett. 2025 Feb 6;16(7):1666–72. doi: 10.1021/acs.jpclett.4c03487 (PMC11849030; doi:10.1021/acs.jpclett.4c03487)
Supplement: Supplementary file 1 — jz4c03487_si_001.pdf [file jz4c03487_si_001.pdf]

Structural Sensitivity of N1s Excitations in N-methylacetamide Solutions: Supplementary Information

E. A. Eronen,[1,*] A. Vladyka,[1] Ch. J. Sahle,[2] and J. Niskanen[1,†]

[1]*University of Turku, Department of Physics and Astronomy, FI-20014 Turun yliopisto, Finland*
[2]*ESRF, The European Synchrotron, 71 Avenue des Martyrs, CS40220, 38043 Grenoble Cedex 9, France*

SUPPLEMENTARY INFORMATION

A. Experimental oxygen K-edge X-ray Raman scattering spectrum of pure liquid NMA

In addition to the nitrogen K-edge, we measured the oxygen K-edge X-ray Raman scattering spectrum for pure liquid NMA at two temperatures 305 K and 350 K. The results are shown in Figure S1 without background removal.

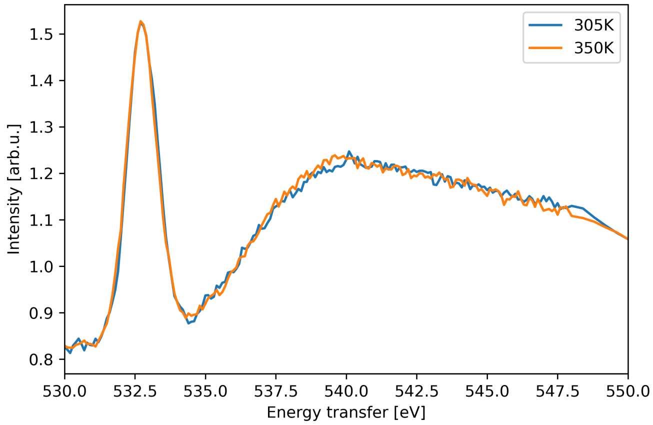

Figure S1. Oxygen K-edge X-ray Raman scattering spectra for pure NMA at 305 K and 350 K without background removal.

B. Molecular dynamics simulation conserved energy drift

We obtained the the conserved energy drifts of our main MD trajectories by a linear fit (in units kJ·mol^{-1}·ns^{-1}·atom^{-1}). These values are presented in Table S1 for each of the eight Tc point.

Table S1. Conserved energy drift obtained from a linear fit.

MNR	T [K]	ΔE [kJ·mol^{-1}·ns^{-1}·atom^{-1}]
pure	305	0.015
	350	0.025
0.33	305	−0.046
	350	−0.004
0.25	305	0.189
	350	0.186
0.20	305	0.203
	350	0.108

* eemeli.a.eronen@utu.fi

† johannes.niskanen@utu.fi

C. The disperse sampling algorithm

Our Monte Carlo inspired disperse sampling (DS) algorithm is defined as follows:

Algorithm S1 The disperse sampling algorithm.

From given N_{pool} points in k-dimensional space ($\mathcal{P}$) select a subset of that consists of N_{subset} dispersely distributed points.
Require: $\mathcal{P}$: A large data pool from which to select the subset.
Require: N_{subset}: The desired number of data points in the subset.
Require: σ_{DS}: scaling factor for the likelihood of a worse outcome being accepted.
Require: stopping condition (e.g. iteration limit, time limit, improvement tolerance etc.).
Randomly select initial subset of N_{subset} points ($\mathbf{P} = \{p_i\}$).
Calculate distance matrix for this subset: $\mathbf{M} : M_{i,j} = d(p_i, p_j)$.
Initialize the current minimum pairwise distance between any two points $d_{\text{min}} \leftarrow 0$.
while stopping condition not met **do**
  Find two points p_k, p_l from $\mathbf{P}$ with the smallest pairwise distance: $d(p_k, p_l) = \min(\mathbf{M})$.
  Pick randomly one of these two points (*e.g.* p_k).
  Pick randomly a new point p_{new} from $\mathcal{P} \setminus \mathbf{P}$.
  Calculate distances $\mathbf{d}_{new}$ from p_{new} to all points of $\mathbf{P}$ except p_k.
  Compare minimum distance with d_{min}, deduce $\Delta = \min(\mathbf{d}_{new}) - d_{\text{min}}$.
  if $\Delta > 0$ **or** $\exp(\Delta/\sigma_{DS}) >$ random value from $[0,\,1)$ **then**
    Replace the old point p_k in the current subset with the new point p_{new}.
    Update the distance matrix $\mathbf{M}$ of the subset.
    Update the minimum distance between any two data points in the subset $d_{\text{min}} \leftarrow \min(\mathbf{M})$.
  end if
  Evaluate if stopping condition is met.
end while
Choose the best found subset (*i.e.* the one with the largest minimum distance between any two points).

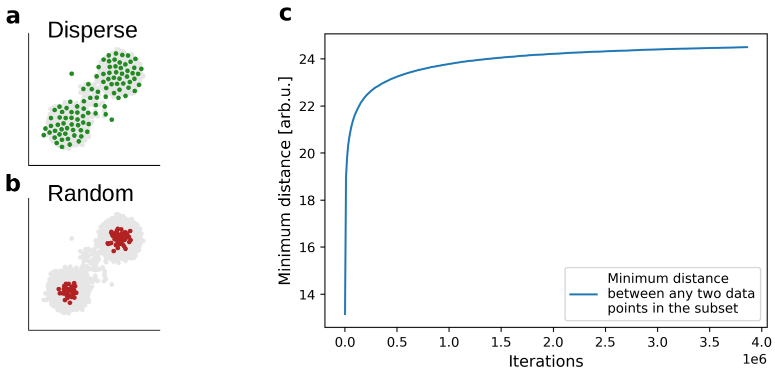

Figure S2. An illustration of the difference between (**a**): disperse sampling and (**b**): random sampling. The former covers the entire available space uniformly whereas the latter is biased towards more dense regions. (**c**): The minimum L_2 distance between any two data points in the best found subset of 10 000 points sampled from the train data pool as a function of the DS algorithm iterations. The improvement quickly slows down which indicates that near-optimal solution can be obtained even without running the algorithm for as long as done here.

The DS algorithm aims to optimize the whole subset simultaneously by iteratively removing the worst point from the subset and replacing it with another one. An illustration of the difference between DS and random sampling is show in Figures S2a and S2b. The procedure requires a suitable metric for comparing the structural data points in the pool. We encoded the atomic number – xyz-coordinate data with a variant of an LMBTR descriptor without a further model selection. We z-score standardized the encoded data and reduced the dimensionality points from the 640 of this descriptor to 100 features with principal component analysis to speed up the algorithm while maintaining more than 98 % of the structural variance. We used the L2 norm to define the pairwise distances $M_{i,j}$.

For the dispersely sampled train data we chose $N_{\text{subset}} = 10\,000$ points from a pool of $N_{\text{pool}} = 1\,280\,000$. We used $\sigma_{DS} = 0.1$ and we stopped the algorithm after 3 860 000 iterations, at which point the results no longer significantly

improved as shown in Figure S2c. For the dispersely sampled early stopping data we chose $N_{\text{subset}} = 1\,000$ points from a pool of $N_{\text{pool}} = 102\;400$ points and we stopped the algorithm after $10\,000\,000$ iterations.

D. The local many-body tensor representation algorithm

In this work, we encoded the structural data using a local version of the many-body tensor representation (LMBTR) [1]. Our implementation, inspired by the DScribe package[2] [3], is defined as follows:

Algorithm S2 The LMBTR algorithm.

Encodes atomistic information (xyz-coordinates and atomic numbers Z) into Gaussian-smeared distance distributions from selected center atoms.

Require: Atoms $\mathcal{A}$: center atoms from which to calculate the distance distributions (*e.g.*, excitation site and some of its neighbors).
Require: Species $\mathcal{S}$: list of all the elements for which to calculate the distance distribution.
Require: Broadening σ of the Gaussian function $\text{Gauss}(x;\mu,\sigma) := \frac{1}{\sigma\sqrt{2\pi}} \exp\left[-(x-\mu)^2/2\sigma^2\right]$.
Require: $\text{grid} = \{d_{\min}, d_{\max}; N\} := \underbrace{d_{\min}, \cdots, d_{\max}}_{\text{N points}}$ on which the LMBTR vectors are evaluated.

for all atom A $\leftarrow \mathcal{A}$ **do**
  for all atomic number S $\leftarrow \mathcal{S}$ **do**
    Get distances $\mathbf{d}^{\text{AS}} = \left\{d_1^{\text{AS}}, \ldots\right\}$ from atom A to all atoms of type S.
    Evaluate $\mathbf{L}_{\text{A}}^{\text{S}} = \sum_i \text{Gauss}(\text{grid}; d_i^{\text{AS}}, \sigma)$.
  end for
end for
Combine all $\mathbf{L}_{\text{A}}^{\text{S}}$ into a single vector: $\mathbf{L} = \bigcup_{\text{A}} \bigcup_{\text{S}} \mathbf{L}_{\text{A}}^{\text{S}}$.

---

1. *Disperse Sampling*

Aided by our prior experience with LMBTR, we used $\mathcal{A} = \left\{\text{N}, \text{H}_\text{N}, \text{C}_2, \text{C}_3\right\}$ as labelled in Fig. 1. For species we used $\mathcal{S} = \{\text{H, C, N, O}\}$. The used grid contained 40 points in the range [0.8 Å, 10.0 Å]. For broadening we used $\sigma = 0.6$ Å. This produced decriptors of size: 4 (center atoms) $\times$ 4 (elements) $\times$ 40 (grid size) = 640.

2. *ROI intensity prediction*

For ROI intensity prediction we used $\mathcal{A} = \left\{\text{N}, \text{H}_\text{N}, \text{C}_2, \text{C}_3, \text{O}\right\}$, species $\mathcal{S} = \{\text{H, C, N, O}\}$ and minimum grid value of 0.8 Å. We determined the number of grid points, the maximum grid value and the broadening width with a model selection discussed in detail later.

---

[1] Haoyan Huo and Matthias Rupp. Unified representation of molecules and crystals for machine learning. Machine Learning: Science and Technology, 3(4):045017, 2022. doi:10.1088/2632-2153/aca005

[2] Lauri Himanen, Marc O. J. Jäger, Eiaki V. Morooka, Filippo Federici Canova, Yashasvi S. Ranawat, David Z. Gao, Patrick Rinke, and Adam S. Foster. DScribe: Library of descriptors for machine learning in materials science. Computer Physics Communications, 247:106949, 2020. doi:10.1016/j.cpc.2019.106949

[3] Jarno Laakso, Lauri Himanen, Henrietta Homm, Eiaki V. Morooka, Marc O. J. Jäger, Milica Todorović, and Patrick Rinke. Updates to the DScribe library: New descriptors and derivatives. The Journal of Chemical Physics, 158(23):234802, 2023. doi:10.1063/5.0151031.

E. Evaluation of the disperse sampling with a separate data set

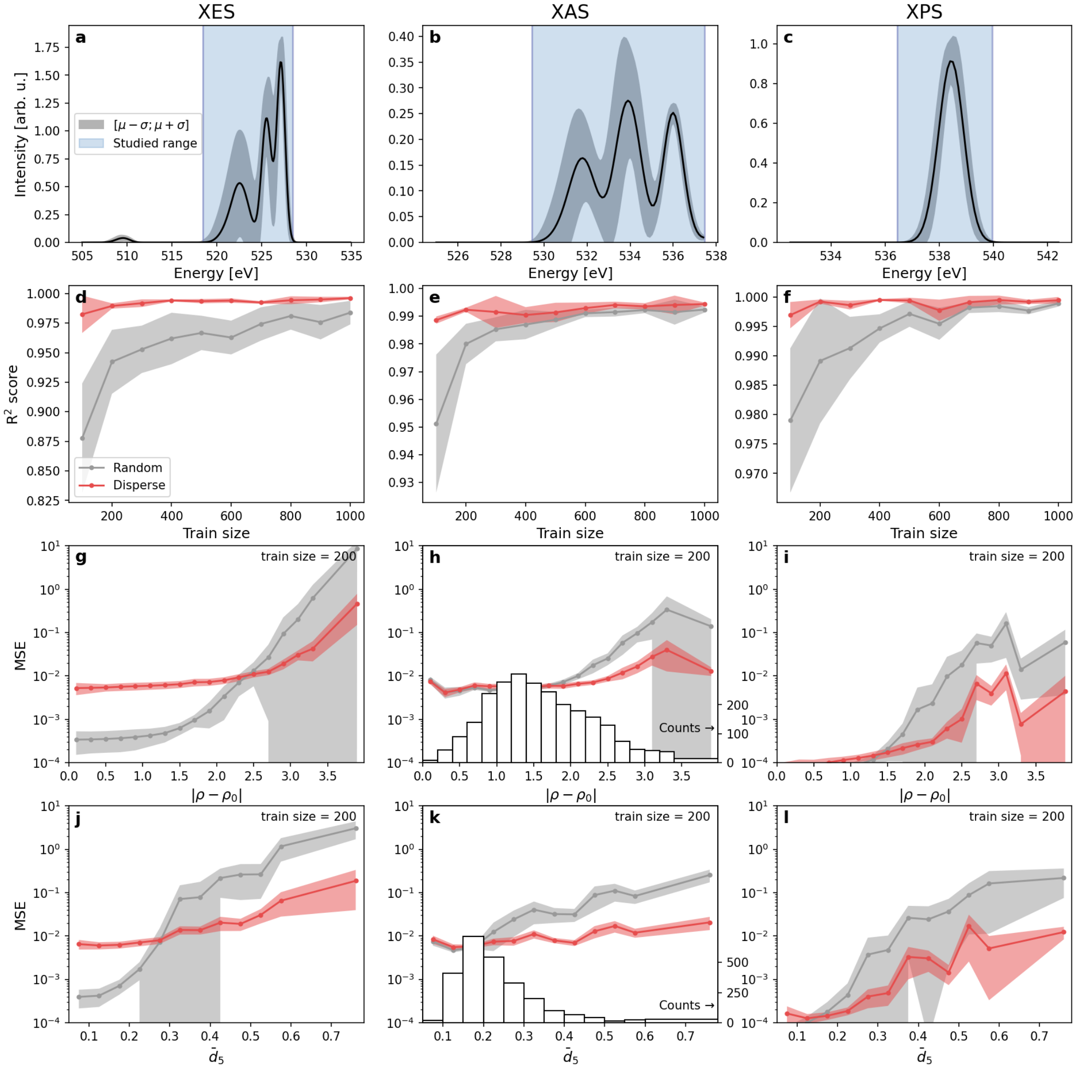

Figure S3. Comparison of the models trained with dispersely sampled data and randomly sampled data for three spectroscopic approaches for simulated water data. **(a–c)** Raw spectral data. Each curve shows a mean spectrum together with $\pm\, 1\,\sigma$ as a grey shaded area. Studied spectral ranges are depicted with blue shaded areas. **(d–f)** Prediction quality measured with R^2 scores for the models as a function of the train size. **(g–i)** Prediction quality measured with MSE for the points binned by their distance from the center of the data set. **(j–l)** Prediction quality for the points binned by the mean distance to their five closest neighbours. Histograms on panels (h) and (k) indicate the number of test points in the corresponding bins. Each line and the corresponding shaded area on panels (d–l) show the mean and standard deviation of 25 independent random or disperse samplings. For details, see text.

To evaluate the disperse sampling approach, we tested the prediction on structure–spectrum pairs of a previously published data for the H_2O molecule[4]. The data consists of 10000 simulated structure–spectrum pairs for X-ray emission spectroscopy (XES), X-ray absorption spectroscopy (XAS), and X-ray photoelectron spectroscopy (XPS). For each spectroscopy, we focused on the ranges depicted in Figures S3a–c. We took 100, 80 and 35 points every

[4] J. Niskanen, A. Vladyka, J. Niemi, and C. J. Sahle. Emulator-based decomposition for structural sensitivity of core-level spectra. Royal Society Open Science, 9: 220093, 2022. doi:10.1098/rsos.220093; data available at doi: 10.5061/dryad.dncjsxm1m

0.1 eV for XES, XAS, and XPS, respectively. The structural data consist of three values for each data point: H–O–H angle and lengths of short and long O–H bonds of the water molecule.

First, the entire dataset was split into train (6500 points), validation (1000) and test (2500) pools. We ran a model selection on subsets of $n = 100, 200, \ldots, 1000$ points evaluating performance on the full validation set. Both spectral and structural data were z-score standardized using the full train pool. Upon model selection, we selected between different width of the neural network (5, 10, 50, 100 or 200 neurons in each hidden layer), number of hidden layers (1–5) and L2-regularization ($10^{-6} \ldots 10^{0}$). Every model was trained with 25 independent random samplings of size n as well as on 25 independent disperse samplings from the training pool. R^2 score was used as a metric while training.

Figures S3d–f shows R^2 scores (evaluated with the validation set) for the best model for each n. Each line shows the mean score for 25 independent samplings; their standard deviations are show as shaded areas. The models trained on the dispersely sampled data consistently perform better than the models trained on the randomly sampled data of the same size. Thus similar prediction quality can be achieved on a considerably smaller training set if it is disperse sampled. Furthermore, models trained on dispersely sampled data are less sensitive to the specific sampling used for the training set compared to the models trained with randomly sampled data.

As seen from the panels d–f, merely 200 dispersely sample train points can be enough to reach convergence of the R^2 score. Therefore, for the further evaluation, we used models trained with 200 points. We introduced two approaches to classify structural data. For the first classification, we calculated the distance $|\rho - \rho_0|$ from each data point to the center of the data set. This metric detects the outliers in data sets which are symmetrically centered around their mean. For the second approach, we used an isolation criteria: the mean distance from the current point to five of its closest neighbors ($\bar{d}_5$). This method allows to qualify the prediction on the points less commonly occurring in a data set. We binned the data points of the test set according to these two criteria, and then evaluated mean squared error (MSE) for the prediction from these bins, as shown in Figures S3g–l. Models trained with randomly sampled points show better prediction quality for more commonly occurring points around data set center. However, models trained with dispersely sampled data points can generalize better for the distant and less commonly occurring points leading to better overall prediction for the entire data set as seen in panels d–f.

F. Spectrum simulation cutoff tests

We chose a random NMA molecule and its local environment at 0.25 MNR concentration and 305 K for spectrum simulation convergence checks. First, we studied the convergence of spectrum shape with respect to the cutoff used to select the other molecules around the absorbing molecule for the creation of the local structures (Figure S4). We included every water molecule fully within r_{H_2O} and every NMA molecule fully within $r_{H_2O} + 2$ Å from any of the atoms of the absorbing molecule. Second, we studied the convergence with respect to the GPAW simulation plane wave cutoff value (Figure S5). We found the spectrum shape to near convergence with $r_{H_2O} = 6.5$ Å ($r_{NMA} = 8.5$ Å) and PW cutoff value of 400 eV, which we chose for statistical calculations. The spectra shown in Figures S4 and S5 were convolved with a Gaussian of a constant full width at half maximum of 0.92154 eV.

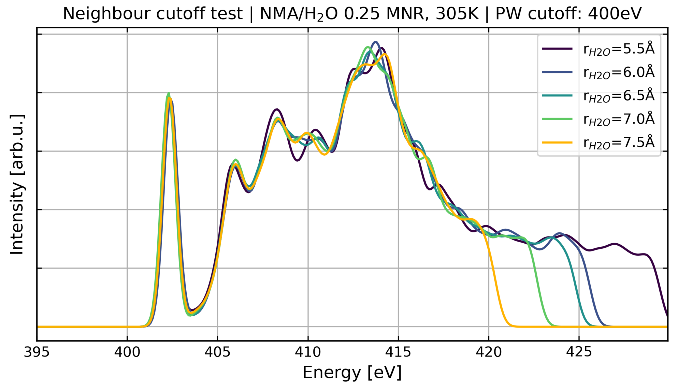

Figure S4. Convergence check for the molecules included in the local neighborhood of the absorbing molecule. Every water molecule fully within r_{H_2O} and every NMA molecule fully within $r_{H_2O} + 2$ Å from any of the atoms of the absorbing molecule was included in the structure used to calculate the spectrum. In these calculations 2500 states were computed instead of 3500 used in the main simulations. We also note that he state limit is reached lower in energy for a larger system.

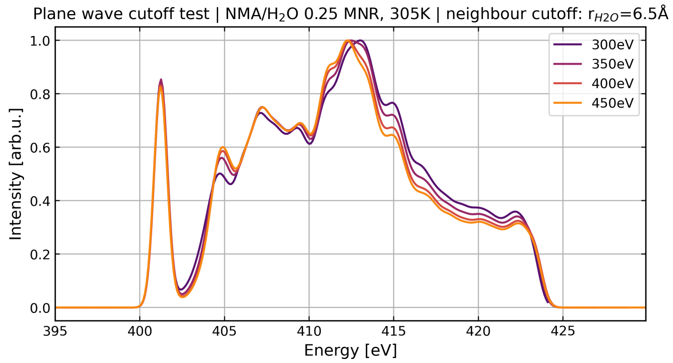

Figure S5. Convergence check for the plane wave cutoff value of the spectrum simulations. We chose $r_{H_2O} = 6.5$ Å ($r_{NMA} = 8.5$ Å) for the simulations. The spectra are shifted so that the pre-peak at around 402 eV match, and scaled so that their maximum value is equal to 1. In these calculations 2500 states were computed instead of 3500 used in the main simulations.

G. The joint hyperparameter search

We ran a joint hyperparameter search[5] of the LMBTR descriptor and Pytorch (version 2.2.1) feed-forward neural network hyperparameters. In this search, we applied five-fold cross validation (CV) while training the model with data points from the dispersely sampled training set (10 000 points). After the model selection we chose the descriptor–model hyperparameter combination with the best CV score and trained the final model with all the training data. In both the model selection and training of the final model we determined the (patient) early stopping with a separate dispersely sampled set of 1 000 points.

Table S2 presents the grid for the joint LMBTR and NN hyperparameter search grid. The best found combination according to the CV score is given in bold. We ran a search spanning the full grid of 107520 hyperparameter combinations. For each trial NN model, we initialized the biases with a constant value of 0.01 and the weights using the Kaiming uniform distribution (nonlinearity = 'selu' with the scaled exponential linear unit (SELU) or nonlinearity = 'leaky_relu' and a = $\sqrt{5}$ with the rectified linear unit (ReLU)). In every case we used the Adam optimizer with learning rate of 10^{-3} (and $\beta_1 = 0.9, \beta_2 = 0.999$) to minimize the R^2 loss (= $1 - R^2$ score).

Table S2. The model selection parameter grid with the best found parameter combination in bold.

	Hyperparameter	Grid points
LMBTR	grid min, Å	{**0.8**}
	grid max, Å	{**3**, 4, 5, 6}
	grid N	{**10**, 20, 40, 80}
	Gaussian width σ, Å	{0.2, **0.4**, 0.6, 0.8}
NN	weight decay	{10^{-8}, 10^{-7}, 10^{-6}, 10^{-5}, 10^{-4}, 10^{-3}, $\mathbf{10^{-2}}$, 10^{-1}}
	number of hidden layers	{1, **2**, 3, 4, 5}
	width of every hidden layer	{8, 16, 32, 64, **128**, 256, 512}
	activation function	{**ReLU**, SELU}
	batch size	{128, 256, **512**}

[5] E. A. Eronen, A. Vladyka, Ch. J. Sahle, and J. Niskanen. Structural descriptors and information extraction from X-ray emission spectra: aqueous sulfuric acid. Physical Chemistry Chemical Physics, 26:22752–22761, 2024. doi:10.1039/D4CP02454K.

H. The first two ECA component vectors for NMA

Figure S6 shows the first two ECA component vectors $\mathbf{v}_1$ and $\mathbf{v}_2$ split to represent all the respective interatomic distances and inverse transformed to absolute scale. Furthermore we divided the element-pair-wise curves by the square of the distance of each feature to its corresponding center.The structure–ROI dependency shows high complexity, as a multitude of features show significant deviation from zero. The bond length to the nearest neighbor atoms of each center always affect N K-edge spectrum, which seen around 1 Å from the respective center. An effect is also often present at 1.5 Å–2.5 Å, which shows that the distance from each center to other atoms belonging to the absorbing molecule affect the spectral regions. In addition, Figure S6d shows a high deviation of H_N–O distribution at around 1.0 Å–2.0 Å.

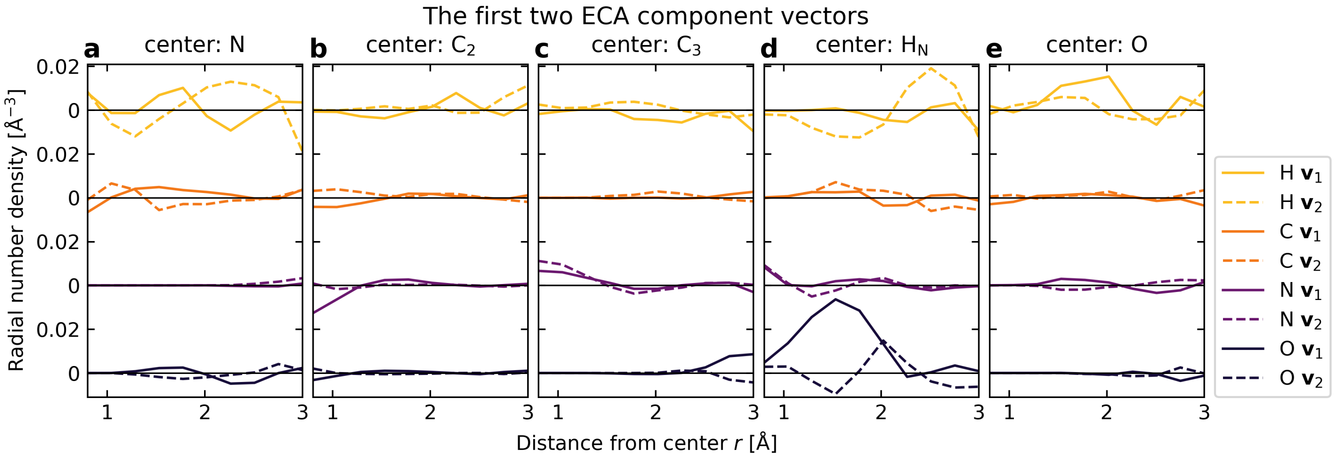

Figure S6. The first and the second ECA component vector ($\mathbf{v}_1$ and $\mathbf{v}_2$) split to represent the different broadened distance distributions within the selected LMBTR descriptor. The vectors have been inverse scaled to absolute structural scale and divided by the squared distance from the respective centers r^2. Generally, a non-zero value implicates that the corresponding feature has an effect on the intensity of one or both of the ROIs.

I. Concentration t distribution max − min for 350 K

Figure S7 shows the difference between ECA expansions of the highest and lowest point of the two dimensional t value histogram in Figure 4f. The results are similar as in the 305 K case.

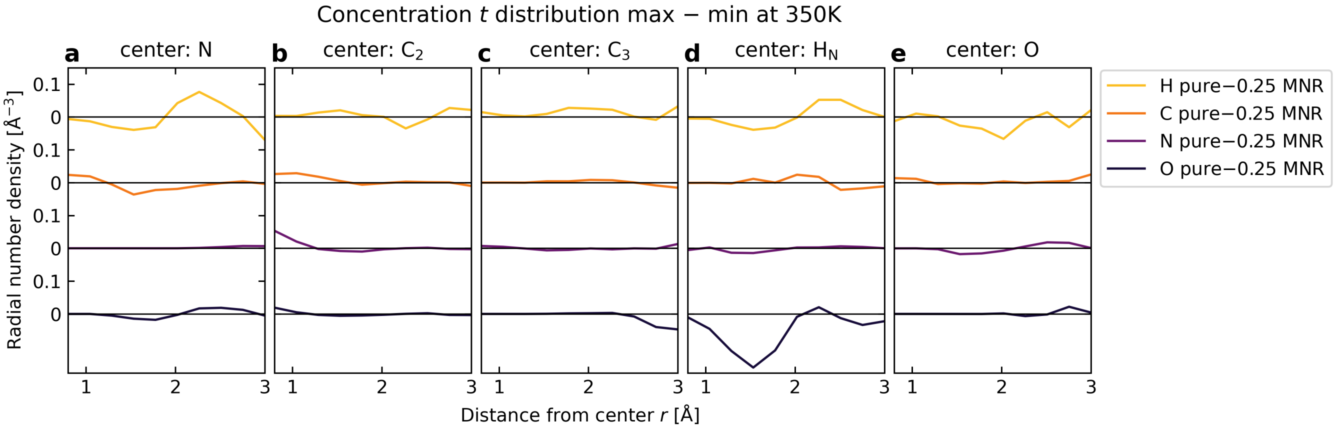

Figure S7. Analysis of the concentration dependency at 350 K, equivalent of that found in the main text for 305 K.

J. Demonstration of the structure-to-spectrum information bottleneck

Not all structural information is reflected in the spectrum, which we demonstrate by choosing two nearly equal local structures in the ECA space and plotting the corresponding structural (LMBTR) vectors. Example 1 has $t_1 = 0.5062$ and $t_2 = 1.1541$, and example 2 has $t_1 = 0.5007$ and $t_2 = 1.1497$. However the corresponding original LMBTR vectors are significantly different as seen in Figure S8.

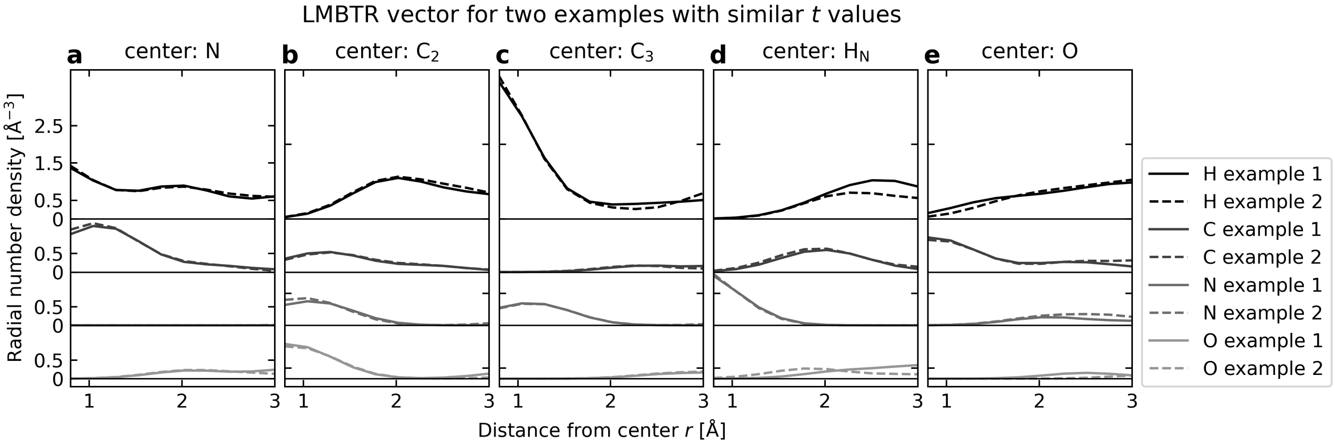

Figure S8. Original LMBTR vectors of two selected structures with nearly identical t values divided by the squared distance to the corresponding center. Even though the spectrally dominant components t_1 and t_2 are very similar, these LMBTR vectors differ significantly.
