# Supplementary material for: Structural Sensitivity of N 1s Excitations in N-Methylacetamide Solutions
Source: J Phys Chem Lett. 2025 Feb 6;16(7):1666–72. doi: 10.1021/acs.jpclett.4c03487 (PMC11849030; doi:10.1021/acs.jpclett.4c03487)
Supplement: Supplementary file 2 — jz4c03487_si_002.pdf [file jz4c03487_si_002.pdf]

jz-2024-03487k.R1

Name: Peer Review Information for "Structural Sensitivity of N1s Excitations in N-methylacetamide Solutions"

First Round of Reviewer Comments

Reviewer: 1

Comments to the Author

This paper describes XAS of N-methylacetamide solutions at N K-edge using X-ray Raman scattering spectroscopy and theoretical analysis. The author's theoretical techniques using the first-principles calculations together with molecular dynamics simulations and the machine learning techniques may be reasonable. Their results agree with the experiments especially below 410 eV for temperature and concentration dependence. Spectral simulation techniques the author's group developed recently may work well.

I propose more explanations for special terms, "z-score standardization". After this modification I recommend this paper will be accepted to the Journal of Physical Chemistry Letters.

Reviewer: 2

Comments to the Author

In this letter, the authors reported nitrogen K-edge spectra of liquid N-methylacetamide and mixture solutions with different water contents at temperatures 305~K and 350~K. There are some significant problems in manuscript organization. It is rather difficult to understand.

(1) Some information given in supporting information should be moved to the manuscript text to make readers easy to read. Thus, an article mode in JPC A/B/C is more suitable for this work.

(2) Figure 3 displayed so many curves in each figure. The size of caption legand is too small to read.

(3) It is also hard to see the curves in Figure 5.

(4) The abstract is too simple. It even did not mention the methods used in this work.

(5) What is the limitation of the method used in this work?

Author's Response to Peer Review Comments:

Please find our detailed response attached. We also provide a marked up version of the revised manuscript displaying all the changes we have made.

January 28, 2025

Dear editor, dear reviewers,

We thank the editor and the reviewers for their time and for their constructive comments. In this letter, we address these comments and list the according changes to our manuscript titled: "Structural Sensitivity of N1s Excitations in N-methylacetamide Solutions".

Reviewer 1

This paper describes XAS of N-methylacetamide solutions at N K-edge using X-ray Raman scattering spectroscopy and theoretical analysis. The author's theoretical techniques using the first-principles calculations together with molecular dynamics simulations and the machine learning techniques may be reasonable. Their results agree with the experiments especially below 410 eV for temperature and concentration dependence. Spectral simulation techniques the author's group developed recently may work well.

I propose more explanations for special terms, "z-score standardization". After this modification I recommend this paper will be accepted to the Journal of Physical Chemistry
Letters.

We thank the reviewer for the encouraging comments and for suggesting this improvement. We have modified the text from:

"To give both equal importance in the analysis, we study the total areas within the computational ROIs after z-score standardization." to:

"To give both equal importance in the analysis, we study the total areas within the computational ROIs after applying z-score standardization. This transformation ensures that the distribution of data points for each feature across the entire dataset has a mean of zero and a standard deviation of one, bringing the two different feature datasets onto comparable scales."

Reviewer 2

In this letter, the authors reported nitrogen K-edge spectra of liquid N-methylacetamide and mixture solutions with different water contents at temperatures 305 K and 350 K. There are some significant problems in manuscript organization. It is rather difficult to understand.

We are sorry to hear the manuscript is difficult to understand, and have done our best to increase its clarity.

(1) Some information given in supporting information should be moved to the manuscript text to make readers easy to read. Thus, an article mode in JPC A/B/C is more suitable for this work.

We appreciate the reviewer's feedback and the suggestion to transfer the manuscript to a regular article format. However, we believe that the current letter format is appropriate for a concise presentation of our findings. This is in agreement with the assessment of Reviewer 1.

We agree with Reviewer 2 that the readability of the manuscript could be improved. To address this, we have added a phrase denoting a change in the perspective of the manuscript.

We modified the text from:

"Some minor contributions can also be traced to changes in distances between neighboring atoms within the NMA molecule.

Instead of using raw atomic coordinates $\mathbf{R}$, descriptors $\mathbf{D}(\mathbf{R})$ are commonly employed to represent structural information in a form that is easily digestible for an ML model."

to:

"Some minor contributions can also be traced to changes in distances between neighboring atoms within the NMA molecule. Next, we turn to the discussion of these results.

Instead of using raw atomic coordinates $\mathbf{R}$, descriptors $\mathbf{D}(\mathbf{R})$ are commonly employed to represent structural information in a form that is easily digestible for an ML model."

We have rewritten the concluding paragraph to better suit the letter form from:

"The nitrogen K-edge excitation spectrum of N-methylacetamide (NMA) manifests notable statistical variation as a function of the underlying local atomistic structure. Changing the temperature or the concentration of the aqueous solution results in an effect at the pre-peak of the spectrum. This behavior is reproduced by classical molecular dynamics together with density-functional-theory-based spectrum simulations. Subsequent emulator-based component analysis of the simulated data revealed that two structural degrees of freedom explain the majority of the spectral variance. In this spectrally decisive subspace the temperature and concentration induced effect in the pre-peak is manifested as a shift within the spread of the structural distribution. The analysis implies that the origin of the spectral effect was mostly hydrogen bonds weakening as temperature or NMA concentration increased, with smaller contributions originating from changes in the interatomic distances in the NMA molecule."

to:

"To summarize, the observed spectral effect is mostly indicative of hydrogen bonds weakening as temperature or NMA concentration increased. This conclusion is based on computations and on

dimensionality reduction into the spectrally decisive subspace of two structural degrees of freedom, which explain the majority of the spectral variance. In this representation, the spectral effect implies a shift in the statistical distribution of the local atomistic structures." and additionally, we rephrased a sentence from: "Consequently, the spectrum remains unaffected."

to:

"In accordance, the ROI intensities for these two structures are similar."

Our submission contained a wealth of technical details that we chose to present in the SI for overall clarity of the letter. In addition, the actual implementation of the analysis scripts and the data are available in Zenodo for the interested. We understand the concern of the reviewer, and have added insights into the summary while keeping the technical details in the SI.

We modified the text from:

"As both the emulator and the descriptor have a wide range of possible hyperparameter values, we ran a joint hyperparameter search [Eronen et al. PCCP 26, 22752 (2024).] and chose the best-performing NN–LMBTR combination."

to:

"The NN and the LMBTR contain numerous hyperparameters to tune. These include e.g. the width, the depth and the weight decay parameter of the NN, and the spatial grid of the descriptor. As each of the hyperparameters has a wide range of possible values, we ran a grid search for them as motivated in Ref. [Eronen et al. PCCP 26, 22752 (2024).] and chose the best-performing NN–LMBTR combination." and from:

"As we demonstrate in the SI, a more ML-friendly training set can be obtained by applying disperse sampling, or a similar method such as the farthest point sampling, on a large pool of MD structures."

to:

"Disperse sampling of structures prior to spectrum evaluation provides a more advantageous training set on a large pool of MD structures (see SI). This procedure generally outperforms random sampling for the spectrum prediction of the H_2O molecule, especially when the data set is small. Disperse sampling was also less sensitive to changes in the selected training data set and generalized better in the sparse regions of the data cloud."

(2) Figure 3 displayed so many curves in each figure. The size of caption legand is too small to read.

We apologise for the flood of information in the figure. Our motivation has been to carry out as comprehensive an investigation as realistically possible. To improve clarity, we have removed the binding energy histograms from the figure. Moreover, we have increased the font size of the legends and edited the caption accordingly. The binding energies presented in the original figure are necessary only for the convolution width and the information is now given in the methods.

We removed the following sentence from the caption:

"The distribution of the simulated N 1s binding energies (BE) are shown as histograms in the respective panels." and modified the text of the methods section from:

"The spectrum simulations produced 3500 energy–intensity pairs for each structure which we convolved using Gaussian functions. The full width at half maximum of these functions equals $0.92154 + \max(0, 6.67 \cdot 10^{-3}(E - E_B))$ in eV, with the N 1s electron binding energy E_B evaluated for the structure. Thus below E_B the width is constant, whereas above E_B the width increases linearly. These general values are based on fitting the sum of the ensemble mean spectra to the sum of their experimental counterparts for the best overall match for all the data. Additionally, the energy scale of the computational results were shifted −1.15 eV."

to:

"The spectrum simulations produced 3500 energy–intensity pairs for each structure which we convolved using Gaussian functions. The full width at half maximum of these functions equals $0.92154 + \max(0, 6.67 \cdot 10^{-3}(E - E_B))$ in eV, with the N 1s electron binding energy E_B evaluated separately for each structure. Thus below E_B the width is constant, whereas above E_B the width increases linearly. These general values are based on fitting the sum of the ensemble mean spectra to the sum of their experimental counterparts for the best overall match for all the data. Additionally, the energy scale of the computational results were shifted −1.15 eV. In this scale, the mean of E_B was 413.9 eV for the whole ensemble-averaged data, and the standard deviation of its distribution was 0.4 eV, respectively." (3) It is also hard to see the curves in Figure 5.

In Figure 5 (now Figure 4) the y-scale of the plot is chosen according to the curve with the most significant deviation from zero. This emphasises the fact that the curves show insignificant values, expected for spectrally irrelevant structural characteristics. This ties to the main point of the manuscript: identification of the decisive structural features. We hope to clarify this aspect by adding the following statement to the caption:

"Values near zero indicate structural features with little to no effect on spectral ROI intensities." (4)

The abstract is too simple. It even did not mention the methods used in this work.

To increase the level of detail, we have modified the abstract from:

"Interpreting X-ray spectra of liquids is complicated by their selective structural sensitivity and ensemble averaging. We report nitrogen K-edge spectra of liquid N-methylacetamide and some of its water solutions at temperatures 305 K and 350 K. The pre-peak in the spectrum shows a shift upon increase of either temperature or N-methylacetamide concentration, an effect our computations reproduce. The subsequent analysis in reduced dimensions, accounting for the involved loss of structural information, reveals that the effect is indicative of weakening hydrogen bonds."

to:

"Interpreting X-ray spectra of liquids is complicated by their selective structural sensitivity and ensemble averaging. We report nitrogen K-edge spectra of liquid N-methylacetamide and its water solutions at temperatures 305 K and 350 K. The pre-peak in the spectrum shows a shift upon increase of either temperature or N-methylacetamide concentration. The effect is reproduced by

our classical molecular dynamics simulations and subsequent spectrum calculations using density functional theory. We apply a data-driven method, the emulator-based component analysis, on the computational data to identify the decisive structural degrees of freedom behind spectral variation. This representation in reduced dimensions accounts for the involved loss of structural information and reveals that the effect is indicative of weakening hydrogen bonds."

(5) What is the limitation of the method used in this work?

We thank the reviewer for pointing out the lack of critical analysis of the applied methodology. We have added a paragraph on the limitations of ECA to the manuscript:

"The validity and accuracy of the ECA method is tied to several factors. First, the accuracy of the simulations is limited by the necessary approximations. We mitigate the associated risk by focusing on ROIs, the intensities of which reproduce an experimental effect. Second, the amount of the available data and its spread within the structural space is restricted, which together with limited model selection cause imperfect emulator performance. Third, the structural descriptor faces several requirements: (i) it must provide accurate ML emulation, and (ii) efficient ECA decomposition, and (iii) be interpretable by a human [Eronen et al. PCCP 26, 22752 (2024).]. Fourth, a higher rank decomposition would increase the covered spectral variance. However, this would happen with diminishing gains and would also add complexity to the analysis. Despite these factors, the applied method provides a mathematical framework for the analysis of the complex spectrum to structure inverse problem via a significant reduction of dimensionality."

Additional changes to the manuscript

In addition to these modifications we have found the following changes to improve the manuscript.

We have spotted an inconsistency in the nomenclature for a Tc point and have changed "T-c" to "Tc" throughout the text. and to improve exactness we replaced a sentence in the methods from:

"The studied NMA/H_2O molecular number ratios 0.33, 0.25 and 0.20 correspond to 57.5%, 50.3% and 44.7% of NMA by weight and the lower temperature of 305 K is slightly above the melting point of NMA at ambient pressure."

to:

"The studied NMA/H_2O molecular number ratios 0.33, 0.25 and 0.20 correspond to 57.5%, 50.3% and 44.7% of NMA (w/w), respectively, and the lower temperature of 305 K is slightly above the melting point of NMA at ambient pressure."

In acknowledgments:

We specified the beamline used at the ESRF:

"at beamline ID20" and replaced a capital "T" to

a lower case one in:

"the Magnus Ehrnrooth foundation"

Non-scientific changes requested by the editorial office

1. Label the TOC graphic as “TOC Graphic”. A caption describing the TOC is notneeded. - Please remove Figure 1 label from “TOC Graphic” and re-label figures and figure citation.

We have fixed the TOC graphic as requested and relabeled rest of the Figures of the main text.

2. Please provide a brief, nonsentence description of the actual contents of each Supporting Information file. - Examples of sufficient descriptions: “1H NMR spectra for all compounds” or “Additional experimental details, materials, and methods, including photographs of experimental setup”. - Examples of insufficient descriptions: ““Figures S1-S3” or “Additional figures as mentioned in the text”.

"Experimental oxygen K-edge X-ray Raman scattering spectra, validation of the structural and spectral simulations, details on the disperse sampling including an evaluation on a separate data set, details on the machine learning hyperparameters and the LMBTR descriptor, additional results obtained with the
ECA method."

3. Please include the URL and date of access for the following incomplete Website reference(s): 11.

We added the date and url to the reference as requested.

On behalf of the authors,
Eemeli A. Eronen

jz-2024-03487k.R2

Name: Peer Review Information for "Structural Sensitivity of N1s Excitations in N-methylacetamide Solutions"

Second Round of Reviewer Comments

Reviewer: 2

Comments to the Author

the present version is recommended for publication.

Author's Response to Peer Review Comments:

Dear Editors,

We thank you for pointing out a typo in the caption of Figure 3. One of the references to panel "c" should have been a reference to panel "b". This has now been fixed, and this is the only change to the main text.

We have also modified the labeling of the SI to match the requested format.

Best regards,

Eemeli A. Eronen
